# A Hybrid Taguchi-Regression Algorithm for a Fuel Injection Control System

**DOI:** 10.3390/s22010277

**Published:** 2021-12-30

**Authors:** Wen-Chang Tsai

**Affiliations:** School of Mechanical and Electrical Engineering, Xiamen University Tan Kah Kee College, Zhangzhou 363105, China; douglas@xujc.com; Tel.: +86-150-6059-7162

**Keywords:** Taguchi-regression algorithm, fuel injection system, direct injection engine

## Abstract

The fuel injection system is one of the key components of an in-cylinder direct injection engine. Its performance directly affects the economy, power and emission of the engine. Previous research found that the Taguchi method can be used to optimize the fuel injection map and operation parameters of the injection system. The electronic control injector was able to steadily control the operation performance of a high-pressure fuel injection system, but its control was not accurate enough. This paper conducts an experimental analysis for the fuel injection quantity of DI injectors using the Taguchi-Regression approach, and provides a decision-making analysis to improve the design of electronic elements for the driving circuit. In order to develop a more stable and energy-saving driver, a functional experiment was carried out. The hybrid Taguchi-regression algorithm for injection quantity of a direct injection injector was examined to verify the feasibility of the proposed algorithm. This paper also introduces the development of a high-pressure fuel injection system and provides a new theoretical basis for optimizing the performance of an in-cylinder gasoline direct injection engine. Finally, a simulation study for the fuel injection control system was carried out under the environment of MATLAB/Simulink to validate the theoretical concepts.

## 1. Introduction

The automobile is popular all over the world, and the automobile industry has become one of the important symbols of a country’s industrialization level, economic strength and scientific and technological innovation ability. Facing the challenges of competitive pressures such as fuel price, emission standards, international technology competition and electric vehicle development, independent development of automotive industry technology is stimulated. With increasing shortage of energy and deterioration of the environment in the twenty-first century, the main goal of direct-injection (DI) engine development is to achieve energy conservation and low pollution [1]. Combustion technology is the basis of an energy-saving and low pollution engine [2]. The fuel injection system is the key module of automotive gasoline direct-injection (GDI) engines. This is the core technical problem that needs to be overcome in the development of GDI engines [3].

The high-pressure fuel injection system of a GDI engine is a complex system with coupling of electric, magnetic, mechanical and hydraulic physical fields. The variation of control and structural parameters of each component has great influence on the normal operation of the whole system. The working characteristics further affect the fuel consumption, power torque and stability of the GDI engine [4]. The establishment of fuel pressure and fuel injection for the high-pressure fuel injection system are separately controllable. According to different operating conditions, the speed and load of the GDI engine are used as the basic signals to achieve its actual working conditions. The operating system follows a fuel map curve with the fuel injection quantity and injection time specified by a pretest, which is used as the basic fuel injection quantity and injection time of the engine. Finally, the water and fuel temperature measured by sensors are used to compensate the fuel injection quantity and injection time to obtain the best fuel injection quantity and injection time to optimize control of fuel injection characteristics such as fuel injection quantity, fuel injection timing and fuel injection rate. At the same time, by considering the influence of various parameters on the performance of direct injection in the cylinder, the high-pressure fuel injection system has become an important means for the GDI engine to reduce pollutant emissions and noise [5].

On the basis of exploring the structure and working principle of the fuel injector, the fuel injector was examined under a rigorous analysis. Through comparative analysis of fuel injection systems [6,7], a testing scheme for the performance of electronic fuel injector was determined. The dynamic response characteristics of the injector and its driver were investigated and a performance test bench established. The real-time fuel injection quantity of the fuel injection system was re-examined and analyzed by the Taguchi method to optimize the performance of electronically controlled fuel injector of engines and to develop a stable and accurate driver [4,8,9,10].

This paper proposes a robust design process to effectively improve each performance index of an electronically controlled injector. First, a sensitivity and contribution analysis of control parameters was carried out to obtain the sensitivity and contribution sequence of these parameters to cycle injection quantity, injection duration and other performance indicators under disturbance conditions; Then, based on the Taguchi method and a regression algorithm, a hybrid Taguchi-Regression analysis for the fuel injection quantity of GDI injectors and a robust optimization calculation of control parameters was implemented to obtain the basic parameter design scheme with good robustness. Finally, a simulation study for a fuel injection control system with an electronically controlled injector was achieved under the environment of MATLAB/Simulink to validate the theoretical concepts. 

## 2. Working Principle

**Figure 1:**
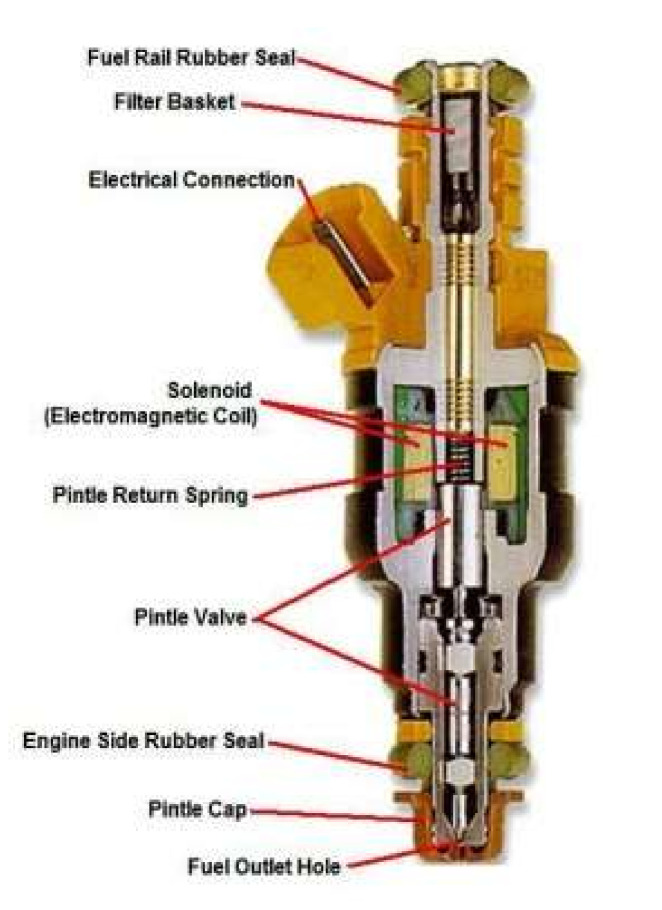
Schematic diagram of an electronically controlled injector.

An electronically controlled injector is mainly composed of solenoid valve, control chamber and needle valve components as shown in Figure 1. When the solenoid valve is not magnetized, the ball valve closes the throttle orifice via the reset spring of the solenoid valve. At this time, the fuel pressure in the control chamber is the same as that in the fuel tank. Since the area of the upper end of the control plunger is larger than that of the control plunger at the fuel tank, the hydraulic pressure on the upper end of the control plunger is greater than the vertical component force on the cone surface of the needle valve at its lower end. Under control of the hydraulic pressure at the upper end of the plunger and the reset spring force of the needle valve, the injector orifice is closed, and the injector stops squirting. As soon as the solenoid valve is excited by the control unit, the solenoid coil is picked up by electromagnetic suction, the ball valve opens with hydraulic pressure, the throttle orifice opens and the fuel in the control chamber begins to flow. When the fuel pressure drops to a certain value, the resultant force between the pressure on the upper end of the plunger and the reset spring force of the needle valve is controlled, being lower than the vertical upward force of the cone of the needle valve at its lower end. At this moment, the needle valve of the injector opens to begin fuel injection. 

### 2.1. Mathematical Model 

The electronically controlled injector is the most important and complex component of a high-pressure fuel system. The control unit sends a signal to energize the solenoid valve while the injector is working. To control the fuel pressure in the chamber, the solenoid valve is excited and then fuel with a certain pressure is injected into the combustion chamber at the most suitable injection time, injection quantity and injection curve. The injector is controlled by the solenoid valve with the help of a hydraulic system to indirectly control the motion of the needle valve. The working process of the injector involves interaction of the mechanical, electromagnetic and hydraulic characteristics of the injector. 

**Figure 2:**
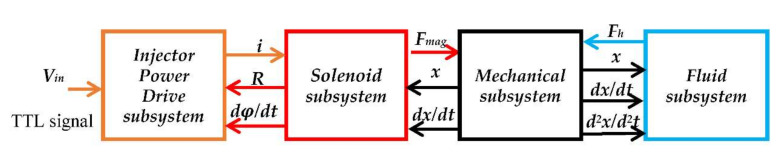
Block diagram of high-pressure solenoid fuel injection system.

The mechanical characteristics of the injector are mainly the reciprocating action of the pointer valve and plunger via the plunger spring. The hydraulic characteristics of the injector depend on the differential pressure generated within the fuel tank, resulting in a certain variation of displacement, velocity, and acceleration of the needle valve when the throttle orifice of the injector control chamber is opened. Electromagnetic force is produced by the starting current through the electromagnetic coil of the injector. As the electromagnetic coil receives the voltage signal sent by the control unit, the armature coil overcomes the preload of the armature return spring, and the armature and ball valve are lifted. The throttle orifice of the control chamber is then opened, and the fuel flow is released. The fuel pressure in the control chamber is reduced, and due to the pressure difference between the fuel tank and the control chamber, the acting force on the needle is greater than the sum of the force of the control chamber fuel pressure and the spring preload. The needle valve rises to open the injector nozzle and the fuel is sprayed out [11]. Variations in mechanical, electromagnetic and hydraulic characteristics of the injector have impacts on the fuel injection quantity, fuel injection consistency and spray characteristics of the injector [12,13]. A schematic diagram of a high-pressure solenoid fuel injection system is shown in Figure 2. An electronically controlled injector is a high-speed solenoid valve. The injection process can be divided into three main processes: needle valve opening, full opening and closing. In the working process, these are subject to the comprehensive action of liquid pressure, spring force, inertia force, friction and electromagnetic force. During the opening process of the needle valve, the electronic control unit sends a turn-on signal to energize the electromagnetic coil and produce the electromagnetic force to overcome the pressure of the spring, fuel pressure, and weight and friction of the magnet assembly. The magnet assembly of the fuel injector is raised to begin fuel injection. During the closing process, the coil is powered off and the electromagnetic force decays rapidly so that the needle valve falls back to the valve seat to stop injecting fuel. According to an analysis of the hydraulic and dynamic characteristics of each component, an electromechanical model of the electronically controlled injector was composed of hydraulic subsystem and moving components [14]. The fuel in the hydraulic tank can be pressurized and the variation of velocity (*v*) of the hydraulic differential valve are based on the following equation:

(1) $$\frac{d\upsilon }{dt}=\frac{SP_{l}-k\left(x-x_{0}\right)-SP_{w}-\xi \upsilon +mg}{m}$$

where *S*—the cross-sectional areas of the solenoid valve; *P_l_* is the line pressure of solenoid; *P*_w_ is the control chamber pressure; *v* is the velocity of the moving part; ξ is the frictional damping coefficient; *k* is the spring constant; x is the displacement of moving part; $x_{0}$ is the valve initially at rest in the bottom-most closed position; *m* is the mass of the moving part, and g is gravity.

When the high-pressure fuel flows in the control chamber through the fuel inlet orifice, the solenoid valve is energized to open the ball valve. At that moment, the high-pressure fuel in the control chamber is squirted through the fuel outlet orifice. The fuel continuity equation of the control chamber controls the amount of fuel $Q_{in}$ flowing into the control chamber. A proportion of fuel $Q_{out1}$ dynamically leaks through the fuel outlet orifice and the other part of the fuel $Q_{out2}$ leaks through the gap of the plunger. The flow equation for the fuel quantity in and out of each vessel can be expressed in the form of:

$$Q_{in}=\varepsilon K_{i}A_{i}\frac{x}{x_{max}}\sqrt{\frac{2\left(P_{l}-P_{w}\right)}{\rho }}$$

(2) $$Q_{out}=Q_{out1}+Q_{out2}=\varepsilon K_{o}A_{o}\frac{z-z_{min}}{z_{max}-z_{min}}\sqrt{\frac{2\left(P_{w}-P_{r}\right)}{\rho }}$$

where $Q_{in}$, $Q_{out}$ is the fuel flow rate into and out of the vessel, *P_l_* and *P_w_* are the line and control chamber pressures respectively; ε is a step function ε = 1 at *P_l_* ≥ *P_w_*; ε = 0 at *P_l_* < *P_w_* ρ is the fuel density, *Ai* and *Ao* are the area of fuel inlet and outlet orifices, and *Ki* and *Ko* are the flow coefficients of fuel inlet and outlet orifices. The flow rate into the control chamber depends on the displacement (*x*) of the hydraulic differential valve with respect to its maximum displacement ($x_{max}$). The flow rate is determined by the ratio of solenoid displacement ($z-z_{min}$) to its maximum displacement ($z_{max}-z_{min}$). The working pressure change in the hydraulic vessel can be given by:

(3) $$\frac{dP_{w}}{dt}=\frac{B\left(Sv+Q_{in}-Q_{out}\right)}{V_{ch}}$$

where the rate of pressure variation ($P_{w}$) depends on the product of the fuel’s bulk modulus (*B*), the cross-sectional area of the movable part (needle) (S), the velocity (*v*), and inversely as the volume of the control chamber ($V_{ch}$). The dynamic characteristics of the moving parts of the fuel spray nozzle (needle) are calculated by the following equation

(4) $$M\frac{d^{2}x}{dt^{2}}+\xi \frac{dx}{dt}+kx=\Sigma F_{m}+\Sigma F_{e}+\Sigma F_{h}$$

*M* is the mass of the movable part (needle); x is the displacement of moving part (needle); ξ is the damping coefficient; k is the spring constant; *F_h_* is the fluid force; *F_m_* is the magnetic force, and *F_e_* is the electromagnetic force. The electromagnetic valve is one of the important components of the fuel spray nozzle. Actuators can be divided into the injector power drive subsystem, solenoid subsystem, mechanical subsystem and fluid subsystem. The simulation model is illustrated in Figure 2. The equations between each subsystem are given below:

(5) $$i=C_{p}\frac{dV_{c}}{dt}$$

(6) $$L_{a}\frac{di}{dt}+(Ra_{\;}+R_{L})i+Vc_{\;}=V_{0}$$

(7) $$F_{h}=fx,\;\frac{dx}{dt},\;P_{w},\;P_{l})$$

(8) $$F_{m}=\frac{\mu_{0}N^{2}i^{2}}{2k_{f}^{2}{\left(\delta_{0}-x\right)}^{2}}A$$

(9) $$\frac{d^{2}x}{dt^{2}}=\frac{F_{m}-k\left(x+x_{0}\right)-F_{h}-\xi \frac{dx}{dt}}{m}$$

where *L_a_* is the solenoid inductance, *i* is the solenoid current; *R_a_* is the solenoid resistance; *R_L_* is the resistance to limit the peak current; *V_0_* is the initial injector voltage across the solenoid, *V_c_* and *C_p_* are capacitor charge voltage and capacitance; *F_h_* is the hydraulic force; *P*_w_ is the control chamber pressure; *P_l_* is the line pressure of solenoid; *F_m_* is the magnetic force; *µ*_0_ is the magnetic permeability in a vacuum; N is the coil number; A is the magnetic flux cross section area; δ is the initial gap; *k_f_* is the magnetic flux leakage coefficient; x is the displacement of the moving part; x_0_ is the valve initially at rest in the bottom-most closed position, and m and ξ are the moving mass and frictional damping constant. 

The needle valve rises rapidly due to the pressure difference increasing. The needle valve lift increases, and the response time of the injector also changes. Thus, the injector injection time, the fuel injection law and the circulating injection quantity are changed. The variation of electromagnetic force affects the establishment of the difference between the injector control chamber and the fuel tank, that is, the electromagnetic subsystem characteristics of the injector affect the hydraulic characteristics. At the same time, the mechanical, electromagnetic and hydraulic subsystem characteristics of the injector are the basis for establishing the injector model. Mechanical, electromagnetic and hydraulic subsystem characteristics are the inherent properties of electronically controlled injectors, which directly affect the fuel injection characteristics of the injector through the interaction between the structures of the injector. The effect of injector structure parameters on fuel injection characteristics is complex. For example, the size of the injector control chamber volume can directly affect the response of the injector; a larger control chamber volume l causes delay of needle valve closing, and negatively affects later atomization of the fuel. If the volume of the control chamber is too small, the needle valve may not reach the maximum lift of the injector, and the flow resistance caused by the injection is intensified, resulting in variation of the injection quantity of the injector.

The minimum start-up pressure of the injector is mainly determined by the flow value of the inlet and outlet orifices in the control chamber and the size of the upper end face of the piston. When the volume of the control chamber and the structural size of the inlet and outlet throttle holes are fixed, the needle valve can reach maximum lift. The injection quantity can be determined when its position is constant [15]. The volume of the control chamber cannot be excessively reduced, and its size should ensure that the needle valve reaches sufficient lift. The inlet and outlet orifices of the control chamber affect the dynamic pressure in the control chamber and the fuel injection relationship. An ideal fuel injection relationship can be obtained by adjusting the size of the inlet and outlet orifices of the injector.

As the actuator of the whole fuel injection system, the electronically controlled injector directly controls the curve shape of fuel injection rate, and affects the injection timing and effective injection pressure. To match the electronic controlled injector, stricter requirements of repeatability and consistency are set for cycle injection quantity, injection duration and injection timing.

### 2.2. Design of an Electronically Controlled Injector Drive

The performance of an electronic control injector is an important parameter in the design of fuel injection control systems, which directly affects the accuracy of the control unit. To further study the electronic control technology of GDI engines, it is necessary to develop devices and equipment that can evaluate and test the characteristics of the electronic control injector. The hardware design of high-pressure fuel injection controller can be simplified by using a new programmable controller for the solenoid valve. The design of a high-pressure fuel injector based on a solenoid valve controller is discussed in [16]. Software can be used to flexibly adjust the parameters of the solenoid valve controller, so as to calibrate the injection quantity of the DI spray nozzle under different parameters to meet the fuel supply quantity of the GDI engine under different working conditions. The related software and hardware design scheme and design details of the high-pressure fuel injection controller have provided engineers with good reference values for further research. At the same time, the experiment also verified the performance of the high-pressure fuel injection controller based on the solenoid valve.

**Figure 3:**
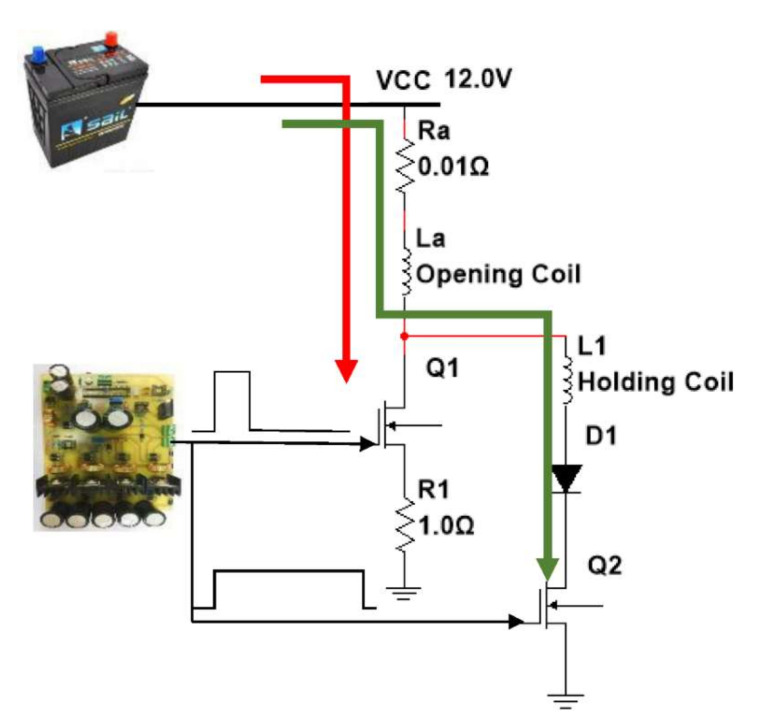
Electrical driving circuit diagram for the HP GDI injector with three pulse width and PWM controller.

**Figure 4:**
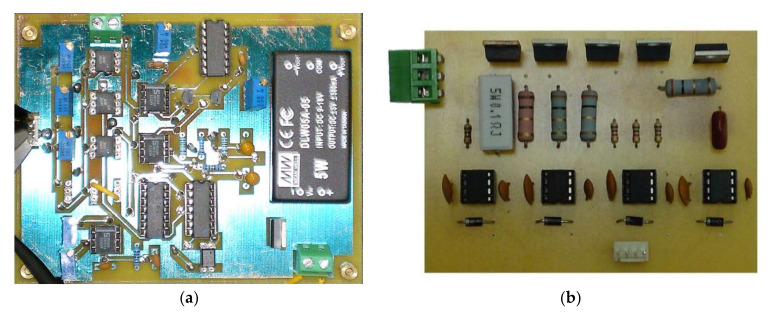
The three-pulse MOSFET injector signal control and driving circuit board. (**a**) Logic operation and driving signal circuit. (**b**) Three-pulse MOSFET driving circuit board.

**Figure 5:**
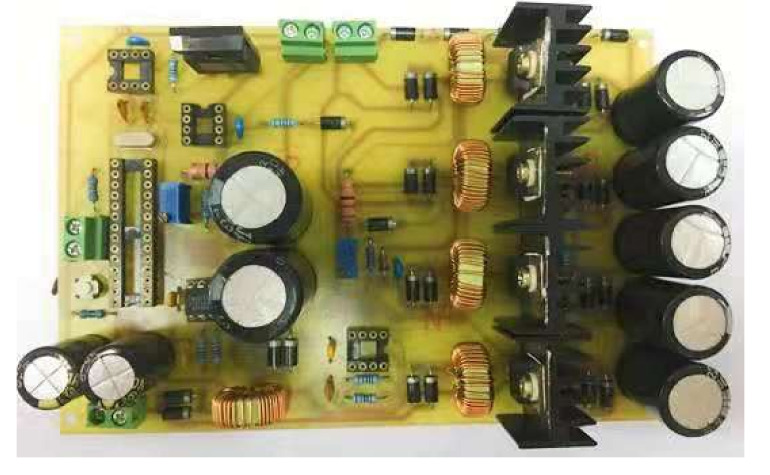
New three-pulse MOSFET injector driving circuit with A DC-Boost Converter.

A stratified mixing region of repetitive position and intake distribution is the key to realizing a combustion system of the GDI engine. The easiest way to achieve stable stratification is to use a separate combustor that provides a good separation area of the mixture. The combustion systems and control strategies of gasoline direct-injection engines from 13 different foreign automobile manufacturers are discussed in detail in [16]. This study investigates the influence of power supply voltages (A), first turn-on injector driving current (B), second injector holding current (C), and injection pressure (D) on the fuel injection quantity. Matlab/Simulink simulation software was applied to design and simulate the injector driving circuit for the requirements of the GDI injector characteristics. The simulation was based on common requirements for automotive applications. The power MOSFET and driver IC models of the three-stage power MOSFETs injector driving circuit is illustrated in Figure 3. As a result, a ready-to-use circuit model was obtained which allows the analysis and the redesign of the power stage later on. Three-pulse power MOSFETs were introduced in the design of the injector driving circuit to carry out the experiments under the operations of high-frequency surge voltages and currents. After the simulation and experimental tests, the improved injector driving circuit with PWM control added into the last pulse duration was required as an actual PCB, as shown in Figure 4. Figure 5 shows the new three-pulse MOSFET injector driving circuit with A DC-boost Converter.

## 3. Design and Analysis of the Fuel Injection System

The electronic control injector is one of the core components in an electronic control fuel injection system. Its performance directly affects the economy, power and emission of the GDI engine. A fast calibration and optimization method of the injection map of the GDI engine based on average model is proposed in [15]. The engine intake model is established for a certain cylinder DI engine by using the average value model, and the data of the model is modified by MATLAB. The mathematical model and simulation calculation for the map curve of fuel injection quantity is established to shorten the development period of an engine electronic control system. It is very important to reduce the cost of the experiment. According to the relevant empirical formula and software, the fuel injection quantity is simulated by using an appropriate mathematical model and reasonable simplification. The fuel map curve of the GDI engine is obtained simply and quickly. The study shows that the method is simple and effective, and the development of an electronic fuel injection system is greatly shortened. The injection performance of the injector is an important parameter in the design of the electronic control system, which directly affects the accuracy of the fuel injection quantity. In order to further study the electronic control technology of the GDI engine, it is necessary to estimate its fuel injection characteristics. The testing for injection characteristics of fuel injectors is important for development of electronic control technology for the GDI engine, and provides a theoretical basis for the development of the testing technology of the fuel injection characteristics of the electronic injector. A test bench for the fuel injection characteristics of electronic injector was built, in which the software and hardware implementation scheme of the test technology was studied. On this basis, the intelligent test system for the fuel injection characteristics of the electronic injector was successfully developed [17,18].

### 3.1. Robust Parameter Design

The working principle for the dynamic process of injector fuel injection and the characteristics of the driving circuit was investigated theoretically, which provided a theoretical basis for the development of a flow rate test of an electronic control injector. A test benchmark for flow and spray characteristics of the electronic control injector of the GDI engine was built to complete the design and development of the electronic control unit of the test system, which could control or detect the state of the test system. In recent years, due to the effectiveness of the Taguchi method in improving product quality and optimization characteristics, researchers have been increasingly interested in the Taguchi method for system and design optimization. Therefore, new methods in system optimization and analysis were introduced to solve the more difficult problems in Taguchi method design optimization [7].

**Figure 6:**
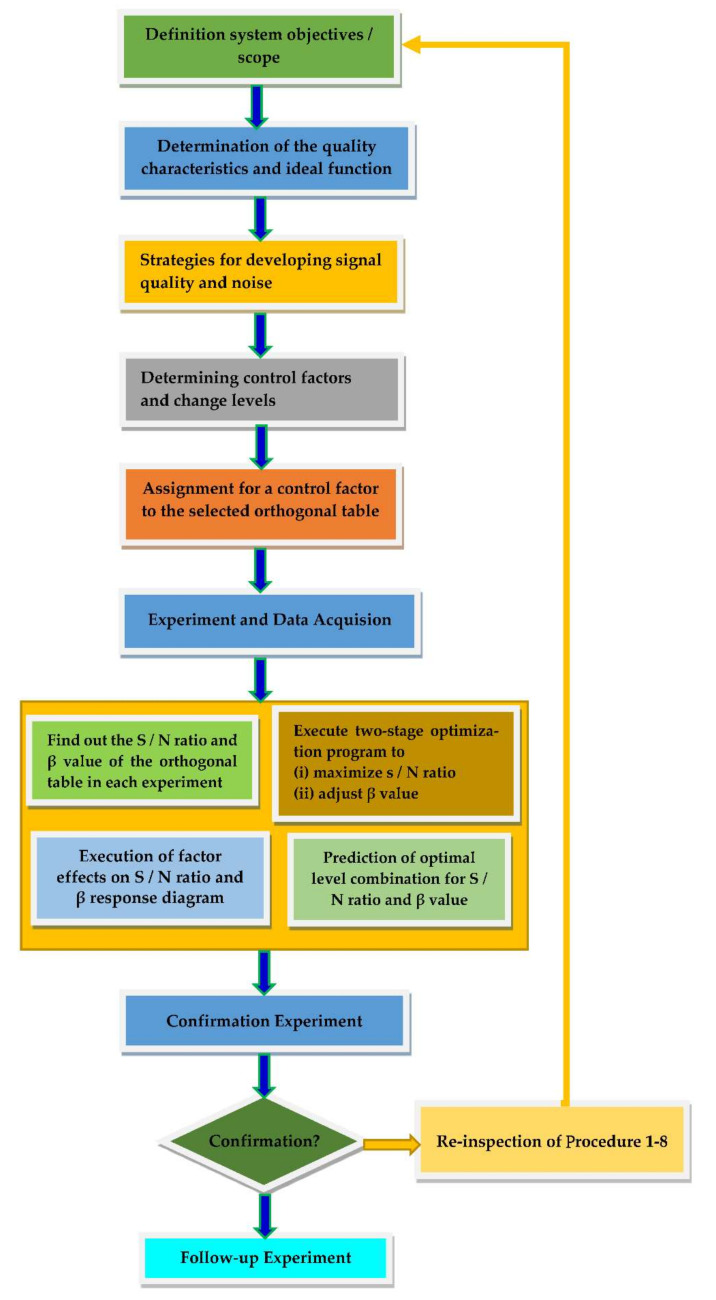
Robust parameter design of a fuel injection system.

This study is based on a new approach that deals with improvement of the optimization process of a high-pressure fuel injection system. With the help of the Taguchi method and its robustness, the aim is to contribute to the development of a more effective optimization method for fuel injection parameters in the injection region of the system. An improved Taguchi method is proposed to solve the design optimization problem, as shown in Figure 6. The specific problem of this study was to overcome the limitations brought by a large number of solutions in the Taguchi method. This goal was achieved by using robust parameter design concept to optimize the fuel injection system. The combination of the Taguchi method and robust parameter design with fewer components can provide better parameter values for design optimization problems. The fuel injection system must have a solution that is less sensitive to parameter variations in order to achieve the design injection quantity closest to the target. The improved Taguchi method is a reliable design method, which uses many ideas in statistical experiment design to obtain reliable information about parameters to achieve the optimal distribution closest to the goal. A lot of research work has been done on the optimization of design systems, and many results have been provided showing the influence of the performance of an optimization algorithm on the optimization of the design system [8].

It is considered that the Taguchi method with an exploratory optimization technique to form a new method may be beneficial to the optimization design for the fuel injection system. This method uses the search ability with improved convergence speed to achieve global optimization. Recently, much research has been done to improve the convergence performance of the Taguchi method to solve objective optimization problems. The idea behind the new method is that the strength of one algorithm can improve the performance of dealing with constraints. In this paper, the parameter design of a hybrid Taguchi-regression algorithm is proposed to solve the optimization problem of fuel injection parameters. The effectiveness of the new method is illustrated by evaluating the problems of the injection system of a GDI engine. In the development of a high-pressure injector driving circuit, due to the limitation of the characteristics of electronic components, the switch assembly must be operated to withstand the impact of high voltage and high current when designing the circuit. A metal oxide semiconductor field effect transistor (MOSFET) and insulated gate bipolar transistor (IGBT) are applied to replace the power transistor for the high-pressure fuel injection system test. The hybrid Taguchi-regression algorithm is used to optimize the operation parameter settings of the fuel injection system and the circuit design of its driving module. The fuel injection pressure is controlled at 8–10 MPA, and the Taguchi experiment design method has three steps.

Step 1: Select the control parameters. In the experiment, two injection MPCI modes are adopted. The first injection time and the second injection time are used as the control parameters. 

Step 2: Three levels are selected for each control parameter. According to the pre-experiment results, to control the maximum pressure rise rate and ensure combustion stability, while taking into account combustion efficiency, the three levels of each variable are selected as shown in Table 1. 

Step 3: the experiment is designed according to the control parameters (A, B, C, D) and levels (1,2,3) as shown in Table 2, in which predicted values of S/N ratio and β for the original factory and new designs are compared. After completing the experiments in the experiment design table, confirmation experiments for the original factory and new designs can be obtained, as in Table 3. At the same time, combined with the analysis of variance method, the F distribution value of injection time to fuel injection quantity can be obtained, with calculation formulas as shown in Equations (10)–(12):

(10) $$\;\bar{y}=\frac{1}{n*m}\overset{n}{\underset{i=1}{\sum }}\overset{m}{\underset{j=1}{\sum }}y_{ij}$$

(11) $$SS_{i}=\overset{m}{\underset{j=1}{\sum }}{\left(\bar{y}-y_{ij}\right)}^{2}$$

(12) $$\mathrm{SS}=\overset{n}{\underset{i=1}{\sum }}SS_{i}$$

where *n* is the number of control parameters; *m* is the number of levels of a control parameter; *y_ij_* is the experiment average result of the *j*-th level of the *i*-th control parameter; y is the average value of experimental results at all levels of all control parameters; *SS_i_* is the sum of variance of the *i*-th control parameter relative to the average result, and SS is the sum of the total variance of the relative mean results of all control parameters. The calculation formula of the *F* value is

*F_i_* = (SS_i_/SS) × 100% (13)

where *F_i_* is the *F* distribution value of the *i*-th control parameter.

### 3.2. Regression Analysis

Regression analysis is often used in interpretation and prediction. The regression equation can be calculated from the obtained samples, and then the influence (contribution) of each independent variable on the dependent variable can be found through the regression equation, to determine the largest influencing variable for statistical and managerial explanation. In the aspect of forecasting, regression analysis is used to predict future changes. Since the regression equation is linear, changes in independent variables cause changes in dependent variables.

Regression analysis can be divided into simple regression and multiple regression. Simple regression is used to explore the relationship between one dependent variable and one independent variable. Regression or multiple regression is used to explore the relationship between one dependent variable and multiple independent variables; its expression is as follows:

1. Simple regression expression:

Y = β_0_ + β_1_X_1_ + ε (14)

β_0_: constant; β_1_: the regression coefficient; ε: error.

2. Multiple regression expression:

Y = β_0_ + β_1_X_1_ + β_2_X_2_ + …. + β_n_X_n_ + ε (15)

β_0_: Constant; β_1_ ….. β_n_: regression coefficient; ε: error.

There are four basic statistical hypotheses in a regression analysis:

1. Linear relationship

The relationship between the dependent variable and the independent variable must be linear, and there is a fairly fixed ratio relationship between the dependent variable and the independent variable. If it is found that the relationship between the dependent variable and the independent variable is nonlinear, it can be transformed into a linear relationship and then regression analysis can be carried out.

2. Normality

If the data shows normal distribution, the error items also show the same distribution. When the number of samples is large enough, the way to check is to use a simple histogram. If the number of samples is small, the way to check is to use a anormal probability plot.

3. Independent variable of error term

Error terms should be independent of each other, that is, there is no correlation between error terms and error terms. Otherwise, the statistical verification power is reduced when estimating regression parameters. Fuel injection quantity can be checked by graphical analysis of residuals, especially the data related to time series and events.

4. The variances of error terms are equal

In addition to the normal distribution of the error term of the independent variable, the number of variables also needs to be equal. The heterogeneity of the number of variables causes the independent variable to be unable to effectively estimate the dependent variable. For example, in the residual distribution analysis, the triangle distribution and diamond distribution can be transformed into the equality of the variables before regression analysis.

5. R^2^ (R square) Determination Coefficient

In this experiment, the coefficient of determination R^2^ is used to explain whether the improvement value is better than the fuel injection quantity recommended by the original factory. The calculation formula is as follows

(16) $$R^{2}=\frac{{\mathrm{SS}}_{t}}{{\mathrm{SS}}_{e}}-1$$

SS_e_: SS of errors; SS_t_: SS of total variations

R^2^ is the variance that can be explained by regression. The total variance from dependent variable y is equal to the variance of regression measurement + SS of errors. The relationship is as follows:

SS_t_ = SS_regression_ + SS_e_ (17)

(18) $$\frac{{\mathrm{SS}}_{\mathrm{regression}}}{\mathrm{SS}}+\;\frac{{\mathrm{SS}}_{e}}{{\mathrm{SS}}_{t}}=1$$

Regression explained variance + total variance of error

## 4. Model Establishment and Experimental Verification

In this paper, the dynamic injection process of a fuel injector is analyzed from two aspects of mechanical motion and electromagnetic force. The opening and closing time of a fuel injector is tested by monitoring the engine air/fuel ratio signal and driving signal of a fuel injector at the same time. The measurement accuracy of the advanced method is high. Based on the analysis of the measured parameters, the performance of the injector is tested, including dynamic and static flow reproducibility, voltage sensitivity test, fuel flow rate test, and so on. The test is repeated many times under the same conditions, and the test results have good repeatability and high detection efficiency. Therefore, the test system has high engineering practical value in the field of fuel injector detection.

### 4.1. Simulation

**Figure 7:**
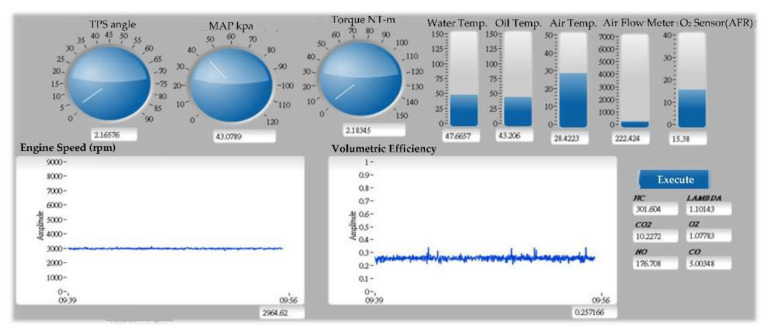
Virtual instrument test platform of the fuel injection control system.

The virtual instrument test platform of fuel injection control system as shown in Figure 7, and allows interaction with the model during simulation. The fuel and air/fuel ratio signals are visualized using dashboard gauges and scopes to provide visual feedback during a simulation run. 

**Figure 8:**
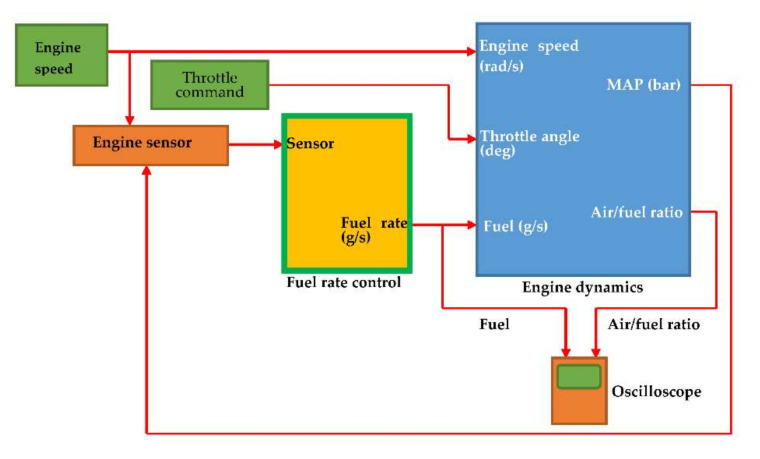
Fuel injection control system.

The fuel rate control block (green frame) of the fuel injection control system as given in Figure 8 uses signals from the system’s sensors to determine the fuel rate which provides a stoichiometric mixture. The fuel rate combines with the actual air flow in the engine dynamics model to determine the resulting mixture ratio as sensed at the exhaust. 

**Figure 9:**
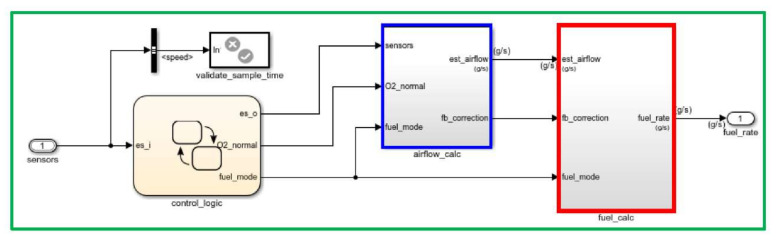
Fuel rate control block.

The fuel rate control block shown in Figure 9 uses the sensor input and feedback signals to adjust the fuel rate to provide a stoichiometric ratio. The model uses three subsystems to implement this strategy: control logic, airflow calculation, and fuel calculation. Under normal operation, the model estimates the airflow rate and multiplies the estimate by the reciprocal of the desired ratio to give the fuel rate. Feedback from the oxygen sensor provides a closed-loop adjustment of the rate estimation to maintain the ideal mixture ratio.

**Figure 10:**
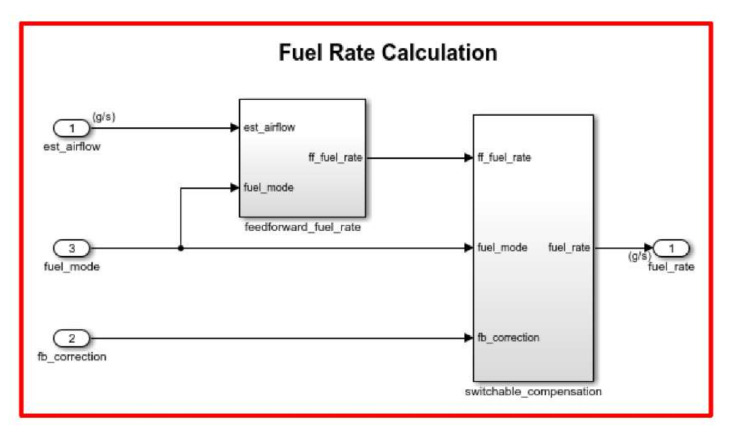
Fuel rate calculation block.

The fuel_calc subsystem (see red frame in Figure 10) within the fuel rate control subsystem sets the injector signal to match the calculation of airflow and fuel injection quantity. The first input is the computed airflow estimation. This is multiplied by the target fuel/air ratio to get obtain the required fuel rate. Normally the target is stoichiometric and equals the optimal air to fuel ratio of 14.6. 

**Figure 11:**
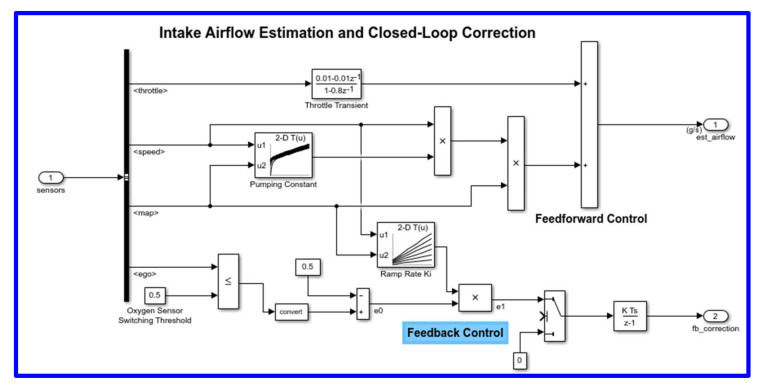
Airflow calculation block.

The airflow calculation block (shown in Figure 11) is the location for the central control laws. This block is found inside the fuel rate control subsystem (open this block with blue bold line). The block estimates the intake air flow to determine the fuel rate that results in the appropriate air/fuel ratio. A closed-loop control adjusts the estimation according to the residual oxygen feedback in order to maintain the mixture ratio precisely. Even when a sensor failure mandates open-loop operation, the most recent closed-loop adjustment is retained to best meet the control objectives.

### 4.2. Test Equipment and Experimental Data Analysis

**Figure 12:**
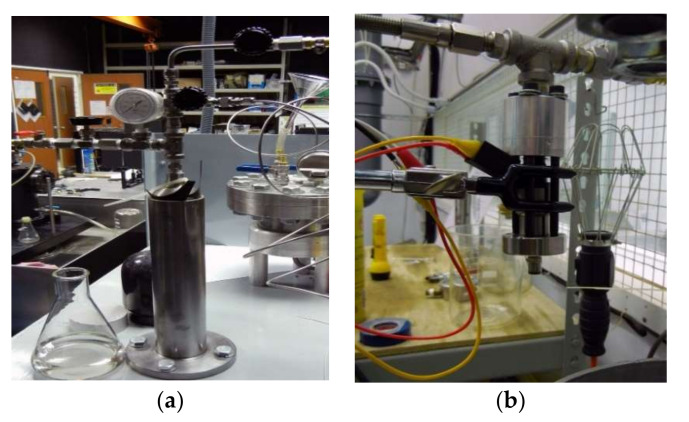
(**a**) High-pressure fuel supply system. (**b**) An electronically controlled injector.

**Figure 13:**
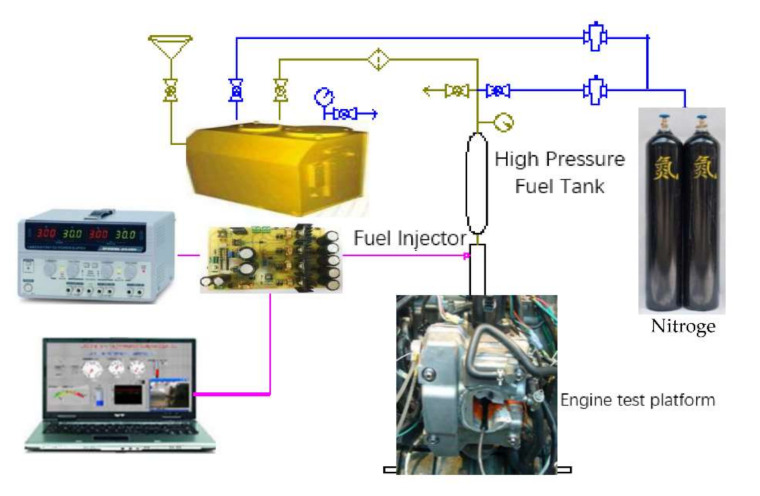
Experimental high-pressure fuel injection.

To avoid problems in the operation of the high-pressure fuel pump for the in-cylinder direct injection nozzle, the high-pressure fuel supply equipment system is used, as shown in Figure 12a,b, which pressurizes the high-pressure nitrogen into the seamless stainless steel cylinder, and uses the regulating valve of nitrogen pressure to push the fuel injection pressure to the operating pressure of 8–10 MPa. In order to verify the rationality of the numerical model, the test-platform is controlled. The parameter settings are as follows: injection pulse width 1.0–2.0 ms, motor speed 1500 rpm, fuel temperature 30.5 °C, injection times 1000 times, injection frequency 10 Hz, and line pressure of 8–10 MPa. The above tests only consider the main injection test to measure the corresponding injection rate. In the same control, a Matlab/Simulink simulation model is run under the control parameters, and the fuel pressure is 8–10 MPa. Comparison is made between the simulation value and test value of injection rate corresponding to 10 MPa in the experimental high-pressure fuel injection system (see Figure 13). Through the comparison of experimental and simulation data, the constructed high-pressure fuel injection system is proved to have superior performance. The numerical simulation model can accurately predict the fuel injection characteristics of the system.

## 5. Results

In the experiment, the engine speed was fixed at 1500 rpm, IMEP is 0.8 MPa and 1.2 Mpa. The experiment objective was to optimize the twice injection time and injection ratio by using a Taguchi experiment design method under this working condition. This was combined with the analysis of variance (ANOVA) method to analyze the contribution rate of twice injection time and injection ratio to fuel injection quantity. In order to reduce Nox emission, EGR rate was 25% when IMEP was 0.8 and 1.2 Mpa, and the air inlet pressure at Mpa was 0.15 Mpa and 0.18 Mpa respectively. The fuel injection pressure was controlled to 8–10 Mpa. 

**Figure 14:**
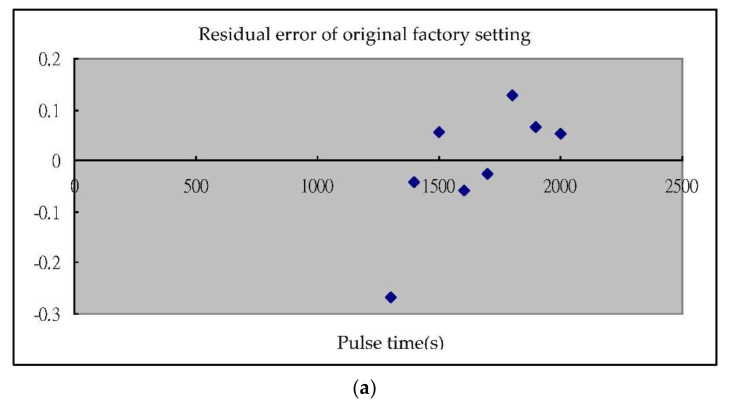

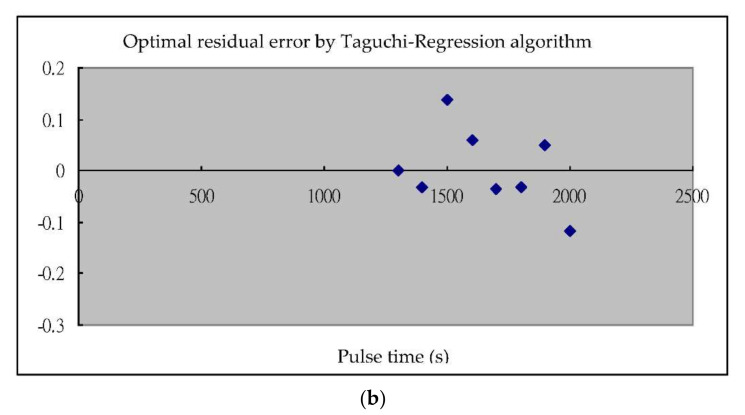
(**a**). Residual error of factory setting for the fuel injection test; (**b**). optimal residual error of injection test by the Taguchi-Regression algorithm.

**Figure 15:**
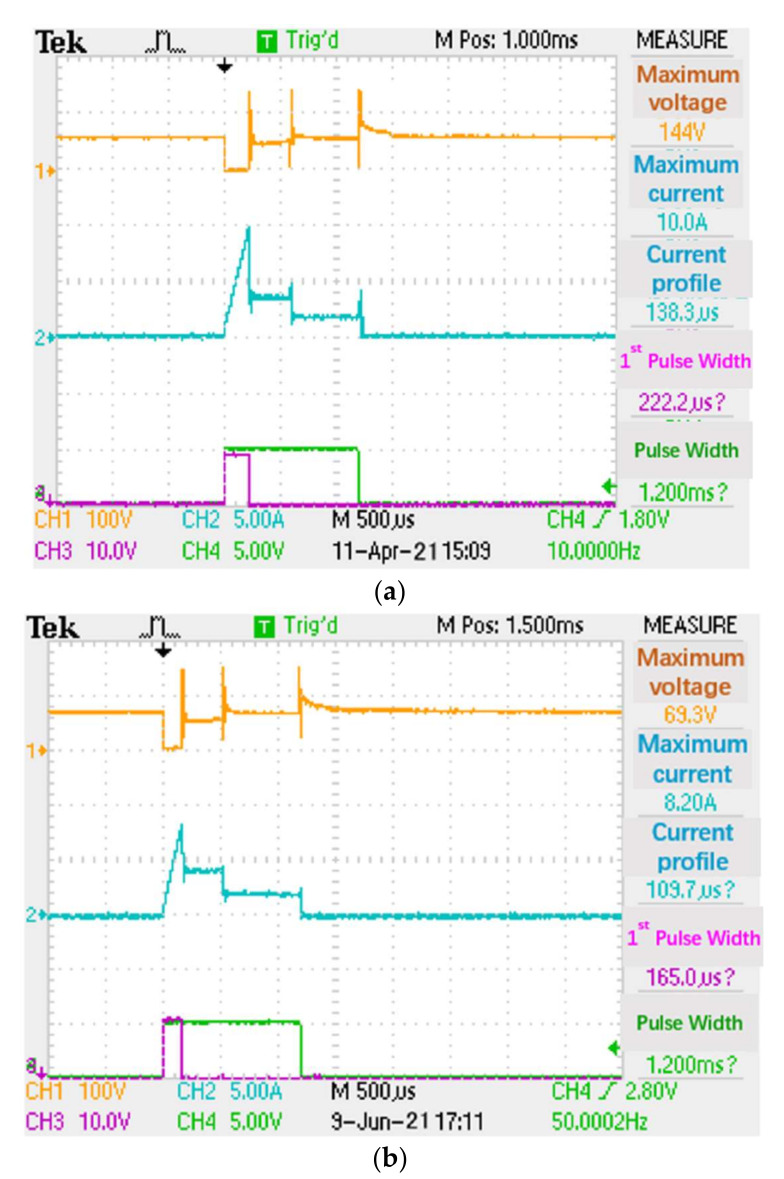
(**a**). Factory setting for voltage and current waveform; (**b**). optimization of voltage and current waveform by the Taguchi-Regression algorithm.

In this study, the Taguchi-Regression method was used in the design of high-pressure injector driving circuit for modifying the experimental control parameters to determine the optimization of designed parameters. The internal circuit adjustment of the injector driving circuit could be improved by using two different designs of circuit architecture. The stability of the circuit was examined for the injection accuracy of a spray nozzle, carefully recording the correction of each fuel injection quantity by adjusting the parameter settings of high-pressure nozzle drive to carry out functional testing step by step. In order to develop a stable operation of the injector driving circuit, the process and its design were improved as follows:

(1) The Taguchi-Regression method was used to find the optimal injection parameters; the structure of this experiment is GDI spray nozzle combined with high-pressure fuel supply system, using experimental bottle and precision electronic scale as the measurement of fuel weight in every 1000 injections. In the study, the experimental design method of direct cross table designed by Taguchi method could effectively list the distribution of all possible experimental results for β Slope calculation, find out the best injection parameter level, and confirm the influence of each control parameter by analysis of variance as well as the final experimental results. During the second Taguchi experiment and the third Taguchi experiment, it was found that the second driving current significantly affected the fuel injection quantity. After analysis, the first current was the starting current. After magnetizing the coil, it was used as the pre-pulling fuel needle. The second current pulled the fuel needle to reach the top of the nozzle, the third driving current PWM operation gradually demagnetized the solenoid valve coil of the nozzle, and closed the fuel needle of the nozzle to stop the injection. Finally, the coefficient of determination R^2^ was analyzed by regression analysis. The injection quantity of the original factory and Taguchi-regression algorithm is compared in the paper. The distribution points of the residual quantity were illustrated and the R^2^ coefficient value improved. From the experimental results it can be seen that the Taguchi-regression algorithm is simple, feasible and effective in the test and optimal design of the high pressure fuel nozzle driver, which can reduce unnecessary experimental tests.
(2) This experiment was aimed at the linear fuel injection setting. The β calculation value was expressed by the calculation formula of S/N noise ratio. The β slope and S/N noise ratio were used for factor response analysis. The S/N noise ratio was used to represent the variation characteristics of the experimental results. The larger the value, the better, which means that the experimental results are less prone to variation and closer to the ideal target position. The β value has no effect on the S/N noise ratio, but the other influential factors on the β value were used to maximize fuel injection quantity.
(3) The simulation results were analyzed by using quadratic regression analysis and regression fittings were carried out to obtain the test parameters and results. By the quadratic polynomial regression model between test indexes, regression analysis was used to analyze the recommended driving power supply used by the original factory settings of the nozzle driver and the best level confirmed by the Taguchi-regression algorithm. In the regression analysis, the setting of the nozzle driver designed and tested by the Taguchi-regression method was higher than the value set by the original manufacturer, as expressed in the R squared value shown in Table 4a,b. This value increased to a certain extent. A diagram shows the variation of fuel injection quantity between the original factory-set value in Figure 14a and the Taguchi-Regression algorithm as shown in Figure 14b. A hybrid Taguchi-Regression algorithm optimized the fuel injection quantity, significantly improving the performance of the nozzle driver.

(4) The Taguchi-Regression method was used to establish the fuel flow rate and air/fuel ratio. The fuel injection responses were obtained by the regression model between fuel flow rate and air/fuel ratio to control the parameters. The influence of response index, comparison of original factory settings and the Taguchi-Regression algorithm for the fuel injection test are shown in Table 5. By parameter optimization, the lift was 0.05 mm and the needle valve lift was 0.34 mm, the fuel injection quantity, the variation rate and response time change rate were 1.70% and 2.27%, respectively. The instantaneous power consumption was also reduced as shown in Table 5 and Figure 15a,b. The optimal settings of the injector driving circuit had less power losses 0.091 W/pulse than 0.148 W/pulse of the original factory setting at maximum voltage 152 V, current 8.2 A, first pulse width 109.7 μs and first conduct width of 165 μs. The original factory and new designs were taken with the settings of A1-B1-C1-D1 and A3-B1-C1-D3 to implement the confirmation experiments. It was observed that variances between two experimental settings presented statistically insignificant differences for the fuel injection quantity. Therefore, two design settings were used for predicted S/N and confirmed S/N to gain the 5.34 db and 5.31 db improvement, as shown in Table 6.

(5) If, at the beginning, the parameter optimization for the fuel injection system of GDI engines is working in an unknown condition, how can we find optimized working parameters? The Taguchi-Regression algorithm provides an important experimental method with good performance. In past experimental research, when we chose working parameter settings for the fuel injection system of the self-designed engine, it was found that the needle of the high-pressure injector sometimes could not continuously and effectively hold, resulting in insufficient injection quantity and engine misfiring in many engine tests. The novelty and contribution of the present paper is to provide improvements on this problem. The Taguchi-Regression algorithm can find the best working parameters of the fuel injection system with a small number of experiments. Next, the optimal working parameter settings in this study had a 5.30 db improvement compared with the original factory setting, which verifies the feasibility for the experimental method using Taguchi-Regression algorithm. Therefore, this research plays an important role in guiding design optimization, matching and calibration of the fuel injector structure.

After S/N and β were analyzed for cross comparison by an ANOVA table, it can be seen from Table 7 that:

1. A and B have the highest contribution after adjusting the voltage and current of the first and second stages to reduce the range of variation.
2. D compares s/N with β. The slope is used to adjust the sensitivity to the maximum value, so the fuel injection quantity is affected when adjusting the fuel supply pressure. In Figure 15a, the original waveform designed by Bosch is used for testing, and shows the pressure change affects the output of nozzle fuel quantity.
3. C is used as a design consideration to reduce cost.

**Figure 16:**
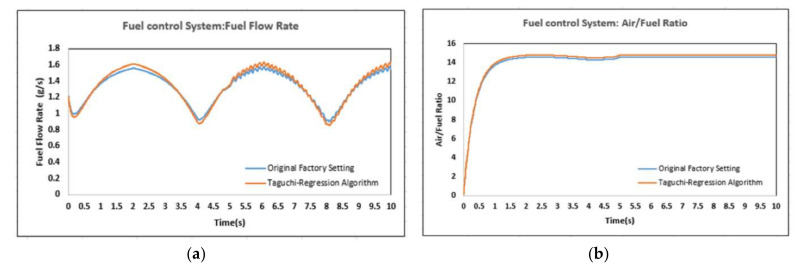
(**a**) Fuel flow rate; (**b**) air/fuel ratio of the fuel injection control system.

In this study, a MATLAB/Simulink simulation model was constructed through the combination of theoretical analysis, simulation and experiment with the working principle and solid structure of the fuel injector. Based on its hydraulic and mechanical process analysis, the original factory setting and Taguchi-regression hybrid analysis were used to carry out an experimental study on the impact of the fuel flow rate and air-fuel ratio indexes of the actual engine test platform to compare the two methods’ parameter settings on fuel flow rate and air-fuel ratio indexes (see Figure 16a,b) and reveal the interaction among these control parameters. The best parameter combinations meeting the injection consistency test are shown in Table 6.

## 6. Conclusions

Aimed at performance and robustness optimization of electronically controlled fuel injectors in a high-pressure fuel system, the following optimization design process was proposed. First, the sensitivity and interaction of key control parameters are analyzed, and the sensitivity ranking of key control parameters on performance indexes such as circulating fuel injection quantity, fuel injection duration, fuel flow rate and air/fuel ratio under known disturbance is given; At the same time, robustness optimization and tolerance analysis are carried out according to each design variable. Then, taking the target quantities for circulating fuel injection quantity as the optimization objective, a basic parameter design scheme with good robustness is obtained by the optimization and calculation of key control parameters. Finally, to further improve the robustness of the design scheme, the machining tolerance range of the above basic design parameters meeting the robustness requirements is adjusted according to the ranking of sensitivity analysis. Through the above process, the design scheme of key control parameters and its correction deviation range can be obtained. The scheme meets the requirements of rapid opening and closing of an electronic control injector, and each target quantity has good robustness, which lays a foundation for the consistency of fuel injection quantity and injection timing of each cylinder. Referring to a high-pressure fuel injection system, the structure and working principles of an electronically controlled injector are studied. Through repeated tests on the dynamic response and flow characteristics of a high-pressure injector, the following conclusions were obtained:

(1) A comprehensive and efficient test scheme for the dynamic response and flow characteristics of an electronic control injector was designed. The software and hardware of the whole test system adopted a modular design, which was convenient for maintenance and development and later functional expansion.
(2) By analyzing the working characteristics, dynamic processes and the test environment of a fuel injector a test system for dynamic responses and flow characteristics of the electronic control injector was built by combining a variety of hardware test equipment with computer software.
(3) The dynamic response and flow characteristics were tested on the constructed test bench. In a perfect test scheme, a large number of tests would be carried out on the high-pressure injector. The test results were analyzed and summarized. Some important parameters such as static flow curve, dynamic fuel flow rate and air/fuel ratio curves in the flow characteristics of the injector were obtained.
(4) To confirm that the experimental mode can accurately describe the fuel injection system, we carried out a confirmation experiment. Table 1 shows the S/N ratio predicted by the experimental mode: 34.7 db in the original design and 40.1 db in the optimal design. Table 2 shows the predicted S/N ratio calculated by performing the original design and new design experiments. Table 3 lists the results of the original design and two settings of new design experiments, and compares them with predictions. The confirmation experimental data of the original design were directly taken from Table 1. The computer simulation experiment under the optimal design shows that the predicted calculated S/N ratio is 34.713 db. It was confirmed that the S/N ratio of the experiment is 40.021, which is consistent with the S/N ratio predicted by the experimental model.
(5) The control parameters A and B had the highest contribution. Adjusting the voltage and current of the first and second stages could be used to reduce the range of variation. The control parameter D compares S/N with β. The slope was used to adjust the zero sensitivity to the maximum value, so the fuel injection quantity is affected when adjusting the fuel supply pressure. In Figure 15a, the voltage and current waveform of the injector driving circuit designed by the original factory (Bosch) was used for testing, and showed the pressure change affected the output of nozzle fuel quantity. The control parameter C was used as a design consideration to reduce the cost of the fuel injection driving system.

## Figures and Tables

**Table 1:** Experimental data with orthogonal arrays and S/N ratios of control parameters for the fuel injection system.

	M = 1000 μs				M = 1200 μs				M = 1400 μs				M = 1600 μs				M = 1800 μs				M = 2000 μs				β	Sd	S/N
L	N1		N2		N1		N2		N1		N2		N1		N2		N1		N2		N1		N2				
	Q1	Q2	Q1	Q2	Q1	Q2	Q1	Q2	Q1	Q2	Q1	Q2	Q1	Q2	Q1	Q2	Q1	Q2	Q1	Q2	Q1	Q2	Q1	Q2			
1	9.82	9.84	9.78	9.82	11.69	11.71	11.64	11.68	13.27	13.29	13.04	13.13	15.28	15.30	15.17	15.24	16.99	16.96	16.99	17.02	18.97	18.98	18.95	18.99	9.50	0.16	35.46
2	10.79	10.80	10.80	10.82	12.84	12.85	12.85	12.87	14.48	14.50	14.54	14.56	16.60	16.63	16.61	16.63	18.39	18.43	18.50	18.54	20.48	20.53	20.55	20.56	10.35	0.23	33.04
3	11.10	11.16	11.15	11.23	13.21	13.28	13.27	13.36	15.08	15.12	15.18	15.20	17.01	17.03	17.23	17.27	19.02	19.05	19.30	19.35	21.13	21.15	21.41	21.46	10.73	0.25	32.78
4	10.70	10.74	10.76	10.76	12.73	12.78	12.80	12.81	14.52	14.54	14.58	14.64	16.76	16.79	16.83	16.86	18.68	18.70	18.83	18.85	20.92	20.93	20.99	21.04	10.48	0.13	38.42
5	10.34	10.33	10.39	10.37	12.31	12.29	12.36	12.34	13.88	13.90	14.06	14.06	15.91	15.95	15.97	16.00	17.68	17.72	17.81	17.84	19.63	19.68	19.72	19.74	9.94	0.22	33.12
6	10.63	10.66	10.71	10.73	12.65	12.68	12.75	12.77	14.53	14.58	14.65	14.68	16.49	16.54	15.59	16.64	18.51	18.54	18.69	18.73	20.44	20.49	20.62	20.66	10.34	0.28	31.27
7	10.44	10.45	10.41	10.42	12.42	12.43	12.39	12.40	14.17	14.18	14.18	14.18	16.30	16.32	16.26	16.29	18.17	18.19	18.18	18.18	20.21	20.25	20.25	20.31	10.15	0.12	38.56
8	10.94	10.96	10.97	10.98	13.02	13.04	13.05	13.07	14.78	14.83	14.90	14.92	17.09	17.13	17.12	17.16	19.10	19.15	19.17	19.24	21.28	21.33	21.33	21.38	10.68	0.13	38.65
9	10.47	10.45	10.45	10.46	12.46	12.44	12.44	12.45	14.24	14.25	14.27	14.29	16.16	16.20	16.20	16.23	18.01	18.02	18.07	18.09	19.89	19.93	19.94	19.97	10.09	0.21	33.54

**Table 2:** Predicted values of S/N ratio and β for the original factory and new designs.

Parameter	Original Design			New Design		
	Setting	Response		Setting	Response	
		S/N	β		S/N	β
A	1	32.6	10.20	3	36.0	10.19
B	1	37.3	10.01	1	37.3	10.01
C	1	34.9	10.17	1	34.9	10.17
D	1	33.7	9.81	3	36.2	10.63
Average value		34.6	10.046		36.1	10.248
Predicted value		34.7	9.530		40.1	10.334

**Table 3:** Confirmation experiments for the original factory and new designs.

Exp.					M = 1200 μs				M = 1400 μs				M = 1600 μs				M = 1800 μs				M = 2000 μs						
					N1		N2		N1		N2		N1		N2		N1		N2		N1		N2				
	A	B	C	D	Q1	Q2	Q1	Q2	Q1	Q2	Q1	Q2	Q1	Q2	Q1	Q2	Q1	Q2	Q1	Q2	Q1	Q2	Q1	Q2	β	Sd	S/N
**Original**	1	1	1	1	11.78	11.79	11.73	11.73	13.41	13.42	13.35	13.34	15.32	15.30	15.28	15.29	17.04	17.03	17.01	17.01	18.93	18.93	18.92	18.91	9.530	0.175	34.713
**New**	3	1	1	3	12.58	12.58	12.52	12.53	14.35	14.35	14.31	14.32	16.54	16.55	16.51	16.52	18.49	18.50	18.48	18.48	20.62	20.62	20.61	20.61	10.309	0.103	40.021
	3	1	3	3	12.75	12.75	12.72	12.74	14.57	14.58	14.61	14.60	16.65	16.65	16.69	16.69	18.72	18.71	18.77	18.76	20.73	20.72	20.79	20.78	10.426	0.117	40.031

**Table 4:** Regression analysis of fuel injection set by the factory, and the Taguchi-Regression algorithm.

Regression Statistics (a)		Regression Statistics (b)	
Multiple R	0.999978737	Multiple R	0.999990760
R^2^	0.999957474	R^2^	0.999981521
Adjusted R^2^	0.857100331	Adjusted R^2^	0.857124378
Standard deviation	0.121580305	Standard deviation	0.077918035

(a) Original factory setting; (b) Taguchi-Regression algorithm.

**Table 5:** Comparison of factory settings and Taguchi-Regression algorithm for the fuel injection test.

	Maximum Voltage	Mean Voltage	Maximum Current	Power Losses	First Pulse Width	First Conduct Width
Factory setting	144 V	59	10 A	0.148 W/pulse	138.3 μs	222.2 μs
Optimal setting	152 V	69.3 V	8.2 A	0.091 W/pulse	109.7 μs	165.0 μs

**Table 6:** Improvement of the original factory and new designs.

	Original Design A1B1C1D1	New Design	Improvement
		A3B1C1D3	dB
Predicted S/N	34.710	40.05	5.34
Confirmed S/N	34.713	40.02	5.31

**Table 7:** Control parameter impact table.

Factor Category	Does it Affect S/N?	Does it Affect β?	Control Factor	Purpose
1	Yes	No	A, B	To reduce the variation
2	No	Yes	D	Used to adjust the maximum sensitivity
3	No	No	C	To reduce costs

## Data Availability

Not applicable.

## Nomenclature

Symbols	Description
DI	Direct Injection
GDI	Gasoline-direct-injection
MOSFET	Metal oxide semiconductor field effect transistor
PWM	Pulse width modulation
PCB	Printed circuit board
Fuel map	Air intake and injection time
MPCI	Multiple premixed control injection
EGR rate	Exhaust gas recirculation rate
IMEP	Indicated Mean Effective Pressure
rpm	Revolutions per minute
S/N	Signal-to-noise dB
ANOVA	Analysis of variance
M	Fuel injection pulse duration (μs)
N1 and N2	Experiments were measured in the morning and the afternoon, respectively.
Q1 and Q2	The first measurement and the second measurement for confirming the precision of the fuel injection measurement system.
